# Mapping European forest archetypes

**DOI:** 10.1007/s13280-025-02318-2

**Published:** 2025-12-03

**Authors:** José I. Barredo, Fernando Santos-Martín, Adrián García Bruzón, Klara Kuželová, Jacopo Giuntoli, Sarah Mubareka

**Affiliations:** 1 European Commission, Joint Research Centre (JRC), Via Enrico Fermi, 2749, 21027 Ispra, VA Italy; 2 Rey Juan Carlos University, Calle Tulipán s/n. 28933, Móstoles, Madrid, Spain; 3 ARHS Developments S.A., Rue Nicolas Bové, 2B, 1253 Luxembourg, Luxembourg; 4 Independent researcher, Via Rosselli, 32, 51016 Montecatini Terme, PT Italy

**Keywords:** Archetype, Europe, Forest, Forest management, Map, Wood

## Abstract

European forests have been extensively altered by human activity. Consequently, comprehensive socio-ecological information on forests is crucial for developing policies from multiple perspectives, such as biodiversity conservation, climate mitigation, and renewable energy. This study presents a map of forest archetypes in Europe by integrating data on both natural and socio-economic characteristics. The map identifies seven archetypes, ranging from natural to intensively managed forests. Our findings indicate that over 50% of European forests are subject to medium to very high use intensity, while the most natural forests comprise only 13% of the total forest area. This forest archetypes map is a valuable tool for informing EU policies, including the EU Biodiversity Strategy to 2030, the Nature Restoration Regulation, and the Bioeconomy Strategy. Understanding the distribution and prevalence of archetypes within regions and countries is essential for assessing the current state of forests and exploring potential management pathways, conservation, and management targets.

**Supplementary Information:**

The online version contains supplementary material available at 10.1007/s13280-025-02318-2.

## Introduction

European forests have been used intensively throughout historical times. As a result, today, only a marginal proportion of European forests remain natural ecosystems, represented by primary forests, without signs of human intervention (Sabatini et al. 2020; Barredo et al. 2025). In addition, approximately 85% of forests are available for wood supply (FOREST EUROPE 2020). Forests in Europe are the result of interactions between ecological processes and human activities over the last six millennia (Kaplan et al. 2009; Schulze et al. 2009), leading to a highly anthropogenic forest landscape where the legacies of profound anthropogenic changes and devastation are still evident today (Poeplau and Don 2013; Mayer et al. 2020).

Although European forest ecosystems are among the most studied in the world, significant gaps remain in our understanding of the interactions between human activities, particularly forest management, and the condition and naturalness of these ecosystems. It is astonishing that the condition (structure and functions) of 29% of forest habitats monitored under Article 17 of the EU Habitats Directive (European Union 1992), which represent around 28% of EU forests (Maes et al. 2020), is unknown, with peaks of 86% in Sweden and 41% in Finland (EEA 2020, 2025b).

There is a growing recognition of the impacts of forest use for the supply of ecosystem services and biodiversity (Senf et al. 2021; Giuntoli et al. 2022; Korosuo et al. 2023). However, a comprehensive understanding of European forests is challenging due to the multiplicity of ecosystem types that have resulted from the interactions between natural processes and human activities throughout history. At this juncture, a complete synthetic picture of these forests is essential for effective policy design and implementation. This is particularly important for enhancing forest resilience and biodiversity, as called for in the EU Forest Strategy to 2030 (European Commission 2021), and for restoring forest ecosystems, as requested in the EU Nature Restoration Regulation (European Union 2024). Achieving these targets necessitates harmonised and synthetic data on forests. The limitations of European National Forest Inventories, due to a lack of harmonisation, further hinder seamless assessments of forests in Europe (Nabuurs et al. 2019; Gschwantner et al. 2024). Actually, the pressing need for harmonised, wall-to-wall forest information for policymakers, scientists, and citizens is the reason behind the proposal for an EU Forest Monitoring Law (European Commission 2023a).

Archetype analysis is a cost-effective approach to characterising and mapping typical patterns that embed both land use intensity and natural features (Levers et al. 2018; Oberlack et al. 2019; Eisenack et al. 2021). Archetype analysis offers an understanding of the characteristics of socio-ecological systems describing general patterns, but detailed enough to account for case–specific particularities (Eisenack et al. 2021; Wingate et al. 2023). Put simply, it is an approach for combining variables representing various natural and socio-economic features into archetypical landscape features. For example, rural typologies have been used to inform agricultural policy (van Eupen et al. 2012; Dou et al. 2021), offering alternatives for identifying comparable rural areas with a certain degree of generalisation. These typologies provide spatially explicit frameworks for scientific analysis and facilitate communication with policy makers and stakeholders.

The use of archetypes facilitates assessing the multidimensional effects of land-use intensity on different biogeographical settings. Archetypes of forests delineate the occurrence of gradients of natural traits vis-à-vis levels of forest use intensity across landscapes, thus providing a more comprehensive understanding of the multiple socio-ecological interactions occurring in forests (Eisenack et al. 2021; Wingate et al. 2023; Barredo et al. 2024). Archetypes of forests can be framed as characteristic patterns of natural traits, forest use intensity, and extent (Levers et al. 2018; Dou et al. 2021), thus delivering a system that embeds the interactions between ecological features and use intensity into representative entities that appear across the land surface (Václavík et al. 2013).

Metrics of land-use intensity are central for archetype delineation due to the transformative capacity of land use as a function of intensity (Václavík et al. 2013; Levers et al. 2018; Dou et al. 2021). There is consistent evidence that land use intensity and landscape structure are determinants of the land use impact on biodiversity and the provision of ecosystem services (Cardinale et al. 2012; Duncker et al. 2012b; Santos-Martín et al. 2013, 2019).

A conceptual archetypal typology of European forest ecosystems that integrates management intensity and naturalness has been proposed by Barredo et al. (2024). This typology describes nine archetypes along gradients of forest management intensity and naturalness. As Barredo et al. (2024) pointed out, management intensity is the main determinant of the level of forest naturalness; that is, there is a close inverse association between management intensity and naturalness. In turn, management intensity is linked to the level of wood production (Duncker et al. 2012a, 2012b), which is expected, as higher net economic returns from timber correspond to greater forestry investments in planting, tending, thinning, harvesting operations, and so on (Duncker et al. 2012b; Pukkala 2016). An inverse association implies that as management intensity increases, naturalness typically decreases. This is because more intensive management approaches often involve altering the forest structure, species composition, and ecosystem processes to optimise wood production, which reduce naturalness. Conversely, less intensive management approaches, such as close-to-nature forestry, tend to preserve or enhance forest naturalness and thus resilience (Gunderson 2000; Thompson et al. 2009). Archetypes of forests provide a useful framework for assessment, with one example being the establishment of certification schemes (e.g. Geraci et al. 2025), among other purposes.

Although there is no shortage of assessments of land system archetypes in Europe (van Eupen et al. 2012; Levers et al. 2018; Dou et al. 2021) or at a global level (Ellis and Ramankutty 2008; van Asselen and Verburg 2012; Václavík et al. 2013), these studies do not specifically address forest archetypes. In fact, in these studies forests are represented by a limited number of archetypes because cropland and urban systems are more prominent. The aim of this study, therefore, is to close this gap by developing a map of European forest archetypes. It seeks to do so by combining variables that describe various forest characteristics, including levels of naturalness, protection, and forest use intensity. This study offers a synthetic socio-ecological understanding of the distribution and accounting of archetypal forests in Europe, both at biogeographical and country levels. The map is expected to be a valuable resource for forest managers, policymakers, and conservation programmes by providing information about the landscape patterns and extent of forest archetypes in Europe. This information will assist them in formulating measures to enhance forest resilience, biodiversity conservation, and restoration. Moreover, the study addresses key research questions concerning European forests, such as determining the proportion of forests under intensive use and identifying their geographical locations. It also explores options for expanding the proportion of strictly protected forests to boost conservation efforts and examines whether current forest use intensity aligns with bioeconomy principles, ensuring the long-term provision of multiple services to society.

## Materials and Methods

As a first step, we surveyed spatially explicit data representing forest naturalness and management intensity characteristics. To identify available data, we used a previous survey on pan-European forest data (Vallecillo et al. 2022) and online data repositories, such as those of the Joint Research Centre of the European Commission, the European Environment Agency, and Copernicus Land Service, as well as peer-reviewed publications. The results of this step revealed a lack of seamless, readily available data on forest naturalness for Europe; therefore, several proxy data sets were collected, describing primary forests, forest protection levels, and forests excluded from wood supply. Observational data on forest management are also lacking at the pan-European level; therefore, as a proxy for forest management intensity, we collected spatially explicit data on forests available for wood supply (FAWS) and forest wood production. The characteristics and sources of the data sets collected are shown in Table 1. The variables on FAWS, forests not available for wood supply (FNAWS), primary forests, and protection levels are nominal variables, while wood production is the only continuous variable.

**Table 1:** Data sets used for the creation of the map of forest archetypes

Archetype factor	Spatial resolution	Reference year	Unit	Source
Forest available for wood supply (FAWS) and forest not available for wood supply (FNAWS)	100 m	2020	FAWS yes/no FNAWS yes/no	Avitabile et al. (2024)
Primary forests	Polygon and point data with minimum mapping unit of 0.5 ha	2000–2019	Yes/no	Kirchmeir and Kovarovics (2016), Sabatini et al. (2020), UNEP-WCMC (2021)
Protected areas	Polygon data	1800s–2024	Protection level categories Ia to VI	Dudley (2008), UNEP-WCMC and IUCN (2024)
Mean wood production	1 km	2000–2010	m^3^ ha^−1^ yr^−1^	Verkerk et al. (2015)

**Fig. 1:**
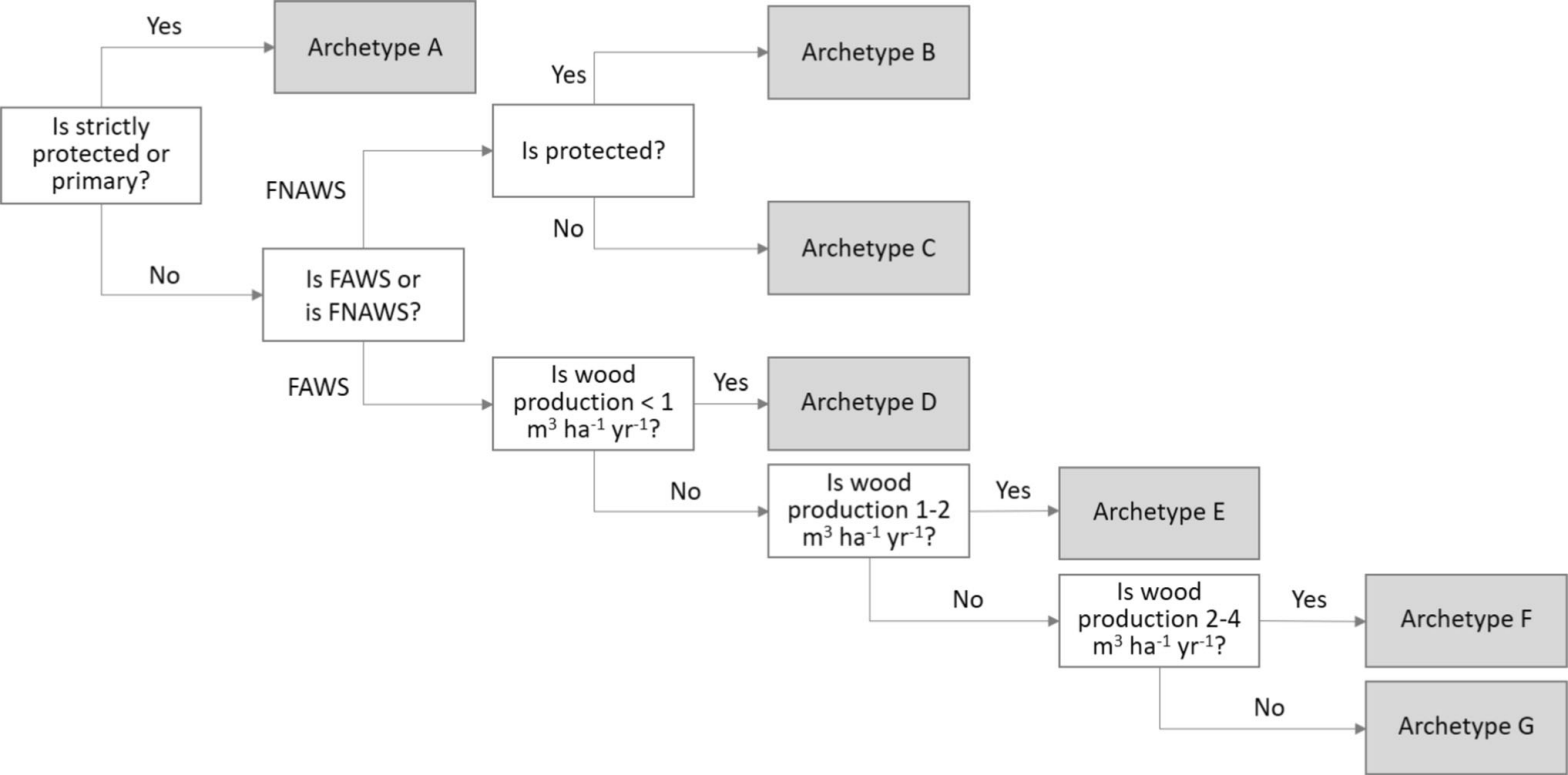
Archetype typology classification system. In grey archetypes A to G: **A** Primary forests and strictly protected forests, **B** Other protected forests, **C** Unprotected FNAWS, **D** Low-intensity forest use, **E** Medium-intensity forest use, **F** High-intensity forest use, **G** Very high-intensity forest use

While some archetype mapping studies use statistical clustering methods to define landscape types (e.g. Levers et al. 2018), others utilise decision trees defined by expert rules and supervised threshold selection (e.g. van der Zanden et al. 2016; Dou et al. 2021). The latter approach builds upon Binary Logic Models, which are well-suited for analysing nominal spatially explicit variables (O'Sullivan and Unwin 2003). Considering that the majority of variables collected are nominal, we used an expert-based archetype classification system. The aim was to create a gradient of naturalness and forest use intensity, focusing on features such as primary forests, protection level, availability for wood supply, and wood production. We employed a classification system to integrate these variables, providing a standardised framework for categorising forest archetypes. The framework is described in Table 2 and was operationalised as shown in Fig. 1.

**Table 2:** Characteristics of forest archetypes in Europe. FAWS: Forest available for wood supply; FNAWS: Forest not available for wood supply. ^a^Other protected forests within FAWS were classified as archetypes D to G based on wood production level. ^b^Unprotected forests within FAWS were classified as archetypes D to G based on wood production level

Archetype	Forest use intensity	FAWS	FNAWS	Wood production (m^3^ ha^−1^ yr^−1^)
A—Primary forests and strictly protected forests	Nature conservation— Passive	Yes	Yes	Not applicable
B—Other protected forests	Nature conservation—Passive	No^a^	Yes	Not applicable
C—Unprotected FNAWS	Nature conservation—Passive	No^b^	Yes	Not applicable
D—Low-intensity forest use	Low	Yes	No	< 1
E—Medium-intensity forest use	Medium	Yes	No	1–2
F—High-intensity forest use	High	Yes	No	2–4
G—Very high-intensity forest use	Very high	Yes	No	> 4

The classification system in Fig. 1 produced a gradient of seven archetypes, ranging from natural forests, represented by primary and strictly protected forests, to forest under very high forest use intensity. This gradient separates forests into two main groups, the first group consists of those exempt from forest use, and the second group includes those oriented towards wood production. The first group is structured into the three archetypes, A–C, representing FNAWS, ordered along a decreasing gradient of naturalness and protection, although archetype A can also occur in FAWS, as discussed below. The second group, consisting of archetypes D–G, represents FAWS and is ordered along an increasing use intensity gradient. We used maps on FAWS and FNAWS derived from Avitabile et al. (2024), to distinguish between archetypes associated with nature conservation and passive use and those under wood production regimes.

FAWS refers to forests where environmental, social, or economic restrictions do not significantly impact the current or potential supply of wood. These restrictions can be based on legal acts, forest management decisions, or of other factors (Forest Europe 2015; Alberdi et al. 2020). Therefore, information on FAWS is essential for delineating the current and potential wood resources available to the wood and timber industry and for bioenergy. In contrast, FNAWS refers to forests that are not available for wood supply; thus, this term includes all forests not considered FAWS (Alberdi et al. 2020). Silvicultural operations are excluded in FNAWS, resulting in forests in a more natural state.

We used the forest/non-forest map, or forest mask, developed by Avitabile et al. (2024), which aligns with national statistics on forest area. The maps of FAWS and FNAWS match the delineation of the forest mask. Next, we describe the implementation of the archetypes according to the classification system in Fig. 1.

### Archetype A: Primary forests and strictly protected forests

This archetype represents the highest level of naturalness. Forests were assigned to this archetype regardless of whether they are in FAWS or FNAWS. This is because, although these forests generally fall within FNAWS, some primary forests outside strict protection and without restrictions may be part of FAWS. However, these forests still remain without signs of human action. This archetype was delineated using data from, firstly, the European Primary Forest Database v2.0—EPFD 2.0 (Sabatini et al. 2020). The UN’s Educational, Scientific and Cultural Organization data set Ancient and Primeval Beech Forests of the Carpathians and Other Regions of Europe, which is listed in the EPFD 2.0, was sourced directly from the custodian due to copyright rules (Kirchmeir and Kovarovics 2016; UNEP-WCMC 2021).

The Food and Agriculture Organization (FAO 2018) definition of primary forest is adopted in the EPFD 2.0 and hence in the present study. These forests have been defined as “naturally regenerated forest of native tree species, where there are no clearly visible indications of human activities and the ecological processes are not significantly disturbed”. The EPFD 2.0 is a geospatial database that harmonises 48 different, mostly field-survey-derived data sets. The database describes 18,411 individual patches represented by polygons and 299 point features, covering an area of 3.7 million ha in Europe, excluding the Russian Federation. The point features, which represent a minor part of the database covering 69,878 ha, are associated with an attribute describing patch area (mean: 315 ha, SD: 2010 ha), except for 78 points that have no data for this attribute. Points with a patch area attribute were represented as proportional circles. For points without this attribute, we used the average patch area of points within the same biogeographical region to create proportional circles. To alleviate major data gaps in Sweden and Norway, we utilised data on potential primary forests in these two countries, also derived from the EPFD 2.0. Data in the EPFD 2.0 are from peer-reviewed scientific literature or based on field checks conducted by trained researchers and professionals, suggesting high data reliability.

Secondly, forests in strictly protected areas were delineated based on data from the World Database on Protected Areas (WDPA) (Dudley 2008; UNEP-WCMC and IUCN 2024). The WDPA is a collaborative project between the International Union for Conservation of Nature (IUCN) and United Nations Environment Programme (UNEP) and is regarded as the most comprehensive global data set on protected areas. The WDPA classifies protected areas into seven categories, ranging from higher to lower protection. We used the first three categories, which are often considered to define strictly protected areas (Hernandez et al. 2021; Cazzolla Gatti et al. 2023): (Ia) Strict Nature Reserve, (Ib) Wilderness Area and (II) National Park. These categories designate areas for biodiversity conservation, where no other activities are allowed (Dudley and Phillips 2006).

Archetypes B and C describe forests within FNAWS, thus forming, together with Archetype A, a group of archetypes under nature conservation or passive forest use (Table 2).

### Archetype B: Other protected forests

This archetype was delineated using the WDPA’s protection categories from III to VI falling within FNAWS. Categories III–VI describe protected areas that allow for multiple uses, including sustainable resource harvesting (Dudley and Phillips 2006). Therefore, we focused on areas within FNAWS in this archetype for excluding forests potentially under wood production regimes. Such forests would instead be classified under archetypes D–G.

### Archetype C: Unprotected FNAWS

Archetype C represents forest areas that are not protected by any conservation designation as defined by the WDPA and are located within areas of FNAWS.

### Forest use intensity in archetypes D–G

Archetypes D–G, representing FAWS, exhibit a gradient of forest use that ranges from low to very high intensity, based on levels of wood production. The data set on wood production provides the mean wood production from 2000 to 2010 across 1 km grid cells (Table 1). The mean wood production in forests within the study area is 1.78 m^3^ ha^−1^ yr^−1^ (SD 1.73 m^3^ ha^−1^ yr^−1^), with minimum and maximum values of < 0.1 and 25 m^3^ ha^−1^ yr^−1^, respectively. The data set exhibits a highly skewed frequency distribution, with the 5th and 95th percentiles at < 0.1 and 5 m^3^ ha^−1^ yr^−1^, respectively. This data set was sourced from Verkerk et al. (2015) and accounts for an array of forest characteristics, including harmonised wood production from national and subnational statistics for 460 administrative units within 29 countries, net annual increment, growing stock, share of the most common tree species, soil, climate, topographic features, forest ownership, and accessibility variables.

The levels of wood production used to define archetypes D–G were based on previous studies and statistics, as described next (Table 2). These levels describe a gradient of wood production that is typically associated with a gradient of forest management intensity in a directly proportional manner (Duncker et al. 2012a, 2012b) and, in turn, with levels of naturalness and biodiversity in an inversely proportional manner (Chaudhary et al. 2016; Gustafsson et al. 2020; Barredo et al. 2024). Generally, more intense management systems, such as even-aged and short-rotation forestry, produce higher yields of commercial species per area and time unit than less intense systems, such as close-to-nature and combined objective forestry (Duncker et al. 2012b). This implies that forests under high wood production regimes are further from a natural state than forests under less intensive wood production regimes and vice versa.

### Archetype D: Low-intensity forest use

This archetype represents forests with wood production below 1 m^3^ ha^−1^ yr^−1^. We adopted this threshold based on definitions by Verkerk et al. (2015), Jonsson et al. (2016), Levers et al. (2018), and Dou et al. (2021), which classify low mean wood production below this value. This threshold often aligns with the natural mortality rate in many European forests, which typically ranges from less than 1% to a few per cent of the standing volume or biomass (Rohner et al. 2012; Lindeskog et al. 2021; Scheel et al. 2022). Therefore, we considered that low-intensity forest use exhibits a maximum wood production close to the level of natural mortality. Apart from this, very low levels of wood production are identified as being below 0.1 m^3^ ha^−1^ yr^−1^ (Verkerk et al. 2015).

### Archetype E: Medium-intensity forest use

Archetypes E and F are two intermediate archetypes between low and very high-intensity forest use. Archetype E corresponds to forest use intensity between 1 and 2 m^3^ ha^−1^ yr^−1^. Forests with wood production in this range, often resulting from multifunctional management approaches (Duncker et al. 2012a), are found in northern Europe and along mountainous regions, such as the southern Carpathians in Romania, the Alps in France and Italy, and the Apennines in Italy (Levers et al. 2014; Nabuurs et al. 2019). This is consistent with Dou et al. (2021), who defined the mean for medium wood production in Europe as 1.5 m^3^ ha^−1^ yr^−1^.

### Archetype F: High-intensity forest use

This archetype includes intensities between 2 and 4 m^3^ ha^−1^ yr^−1^. This range is common in forests under intensive management, such as those in Romania, as well as central and Southern Sweden and Finland (Bergh et al. 2005; Levers et al. 2014; Verkerk et al. 2015; Blujdea et al. 2021). The range adopted is consistent with Dou et al. (2021), who defined the mean for high wood production in Europe at 3.65 m^3^ ha^−1^ yr^−1^.

### Archetype G: Very high-intensity forest use

This archetype describes forests with use intensity above 4 m^3^ ha^−1^ yr^−1^. This amount is often considered the lower bound threshold for very high wood production levels (Levers et al. 2014, 2018; Verkerk et al. 2015). Such levels are often found in forests under intensive even-aged, and short rotation forestry, like those in southernmost Sweden, the Landes de Gascogne region in France, Estonia, Czech Republic, as well as in Switzerland and some areas in northwest Spain and Portugal (Nabuurs et al. 2019).

### Assessing wood production in archetypes D–G

One-way ANOVA followed by the post hoc Tukey HSD test was used to assess statistically significant differences at 95% confidence level in mean wood production among archetypes D, E, F, and G (those using the wood production variable in their delineation) on 1000 randomly sampled grid cells for each archetype, selected to avoid spatial autocorrelation. The one-way ANOVA test is appropriate when comparing more than two groups to identify if at least one group mean is different from the others. Following the ANOVA, the post hoc Tukey HSD test was applied to asses which specific pairs of archetypes have significant differences in their mean wood production.

### Assessing gradients of forest use intensity

To explore whether there is a latitudinal and longitudinal gradient of wood production in forests within archetypes D–G, i.e. those in FAWS, we conducted an assessment of wood production and the distribution of archetypes across nine 500 km latitudinal and longitudinal slices in the study area. We assessed statistically significant differences in wood production among the nine latitudinal and longitudinal slices on 1000 randomly sampled grid cells for each slice, selected to avoid spatial autocorrelation. We employed one-way ANOVA followed by the post hoc Tukey HSD test to assess statistically significant differences at 95% confidence level among the latitudinal and longitudinal slices.

### Assessing archetypes mean patch size

To analyse the landscape structural characteristics of the archetypes, we calculated the mean patch size (MPS) of forests within each archetype across biogeographical regions. Patch size is a simple and intuitive metric for assessing landscape structure. It is among the most important and useful pieces of information contained in the landscape (McGarigal and Marks 1995; Vogt et al. 2022; Riitters and Vogt 2023). We calculated MPS following McGarigal and Marks (1995). First, we identified contiguous groups of more than two grid cells (patches greater than 2 ha) that share the same archetype, using the Moore neighbourhood (eight surrounding grid cells) with the archetypes map at a 100 m grid size as input. Second, we determined the size of these patches. Third, we calculated the MPS for each archetype within each biogeographical region. A one-way ANOVA and the post hoc Tukey HSD test were used to assess statistically significant differences in the logarithm of MPS across archetypes within each biogeographical region. The tests were conducted on 1000 randomly sampled patches for each archetype, selected to avoid spatial autocorrelation. Additionally, Tukey HSD test was repeated iteratively 1000 times on 1000 randomly sampled patches to account for the effects of sampling on the p values. With this information, we considered p values to be significant for those pairs of groups where *p* < 0.05 in at least 95% of the 1000 iterations.

### Spatial domain

The spatial domain of the archetypes map includes the 27 EU countries (except Croatia due to lack of data on wood production), Switzerland, Norway, and UK. However, it excludes the Canary Islands (Spain) and Azores and Madeira Islands (Portugal) due to lack of data. The results are presented at the European, biogeographical region (EEA 2025a) and country levels. The Alpine biogeographical region was split into two subregions because of the significant environmental differences between the Alpine-Scandinavian and the rest of the Alpine zones in more southerly latitudes, following Maes et al. (2023). Therefore, one subregion represented the Alpine-Scandinavian and the other the rest of the Alpine subregion, that is, the mountain ranges of the Alps, Apennines, Pyrenees and Carpathians.

### Sensitivity analysis and benchmarking

We conducted a sensitivity analysis to determine how changes in wood production thresholds might affect the extent archetypes D, E, F, and G (see supplementary material S1). Additionally, we conducted a comparison of the distribution of archetypes across biomes against the results of Nagel et al. (2025), which reported the proportion of strict forest reserves, extensively and intensively managed forests in European countries. Nagel et al. (2025) used data on country-wide silvicultural practices and compiled a database on strict forest reserves across Europe (see supplementary material S1).

## Results

**Fig. 2:**
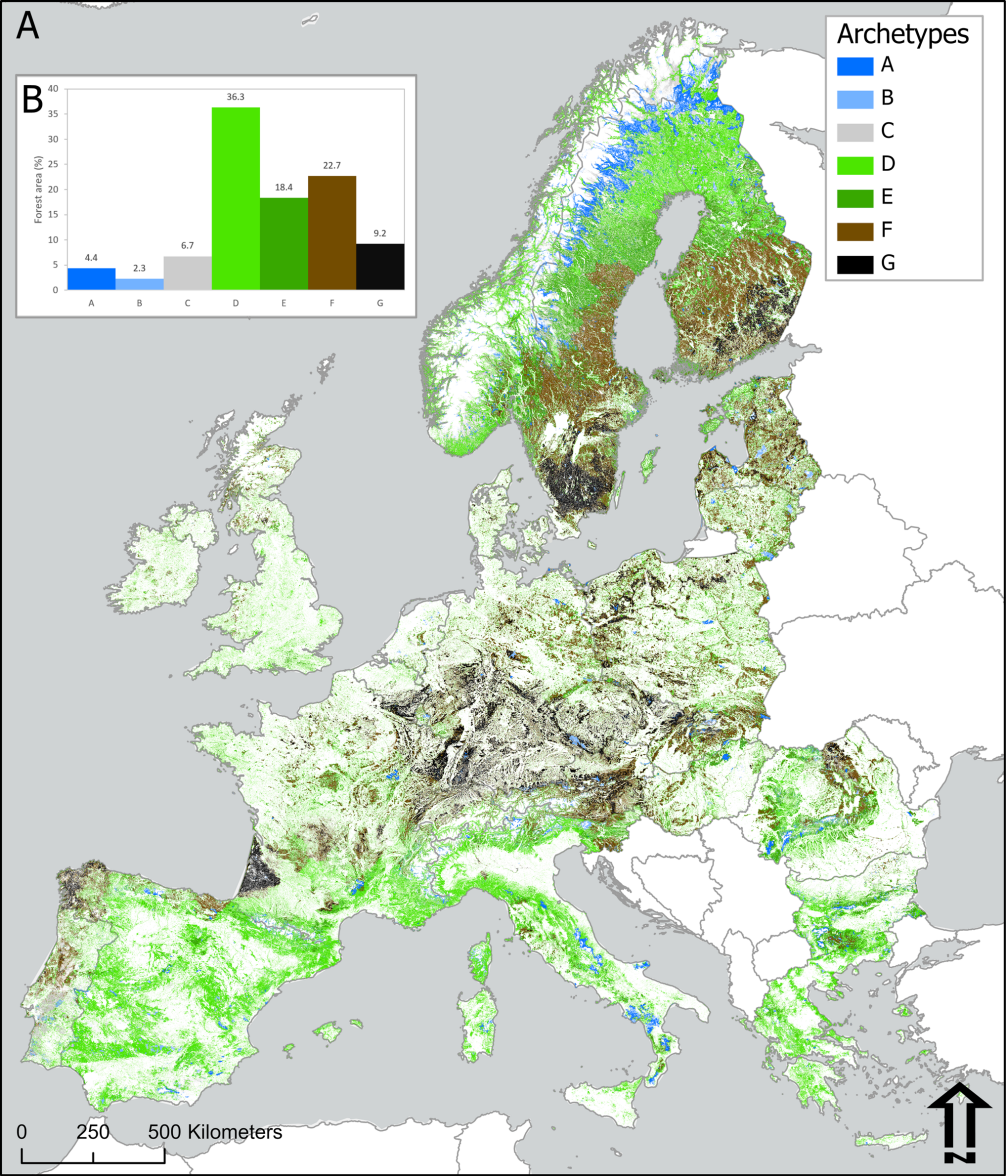
**A** Spatial pattern of forest archetypes in Europe. **B** Frequency distribution of forest archetypes. Archetypes: **A** primary forests and strictly protected forests, **B** other protected forests, **C** unprotected FNAWS, **D** low-intensity forest use, **E** medium-intensity forest use, **F** high-intensity forest use, **G** very high-intensity forest use (data available at Zenodo: 10.5281/zenodo.17486082)

The forest archetypes map delineates the spatial pattern of seven archetypes representative of forests in Europe at a 1 km spatial resolution (Fig. 2A). It depicts a forest extent of over 1.7 million square kilometres across 29 countries. Among the archetypes, archetype D represents the largest proportion of the study area at 36% followed by archetypes F and E, with 23% and 18%, respectively (Fig. 2B). These three archetypes are part of FAWS. Our results indicate that more than half of the forest area, 50.3%, falls within medium to very high forest use intensity archetypes, namely E, F, and G (Fig. 2B). In contrast, the three archetypes representing FNAWS in primary, protected, and unmanaged forests, namely A, B, and C, cover only 13% of the forests within the study area. The remaining 36% of the forest area corresponds to archetype D, which includes forests under low use intensity and other forests potentially available for wood production but likely unused, such as low productivity forests in the Mediterranean region and northern Fennoscandia. The forest area within each archetype is shown in the bottom row of Table 3.

**Table 3:** Extent of forest archetypes per European biogeographical region. Archetypes: Archetypes: (A) Primary forests and strictly protected forests, (B) Other protected forests, (C) Unprotected FNAWS, (D) Low-intensity forest use, (E) Medium-intensity forest use, (F) High-intensity forest use, (G) Very high-intensity forest use. Croatia is not included in this assessment due to a lack of data on wood production. Data for Malta are not considered because it shows no wood production and almost no forest area

Biogeographical region	Total extent (1000 km^2^)	Archetype extent (1000 km^2^)						
		A	B	C	D	E	F	G
Alpine	162	5.2	12.9	14.8	42.5	31.4	41.6	13.7
Alpine-Scandinavian	85	24.8	1.1	26.3	27.0	5.4	0.2	0.0
Arctic	0.1	0.02	0.001	0.05	0.03	0	0	0
Atlantic	182	2.1	1.6	12.8	86.6	31.3	32.0	15.8
Black Sea	3	0.1	0.2	0.1	0.7	1.4	0.03	0
Boreal	579	30.0	4.9	42.5	127.1	136.6	190.5	47.8
Continental	391	4.9	6.5	4.2	100.9	82.6	111.2	80.9
Mediterranean	303	7.6	12.1	15.9	236.3	20.4	9.9	0.7
Pannonian	25	0.7	0.4	0.2	7.9	8.1	7.2	0.9
Steppic	2	0.1	0.04	0.01	0.9	0.9	0.3	0.0
Total	1733	75.5	39.8	116.9	630.0	318.1	392.9	159.9

The map in Fig. 2A shows that the archetypes depict large landscape patterns across the continent. For example, archetype D occurs in large zones of the Mediterranean region in Spain, southern France, Italy, and Greece, while a large block in the southernmost part of the Scandinavian Peninsula in Sweden corresponds to archetype G.

### Archetypes A and B

**Fig. 3:**
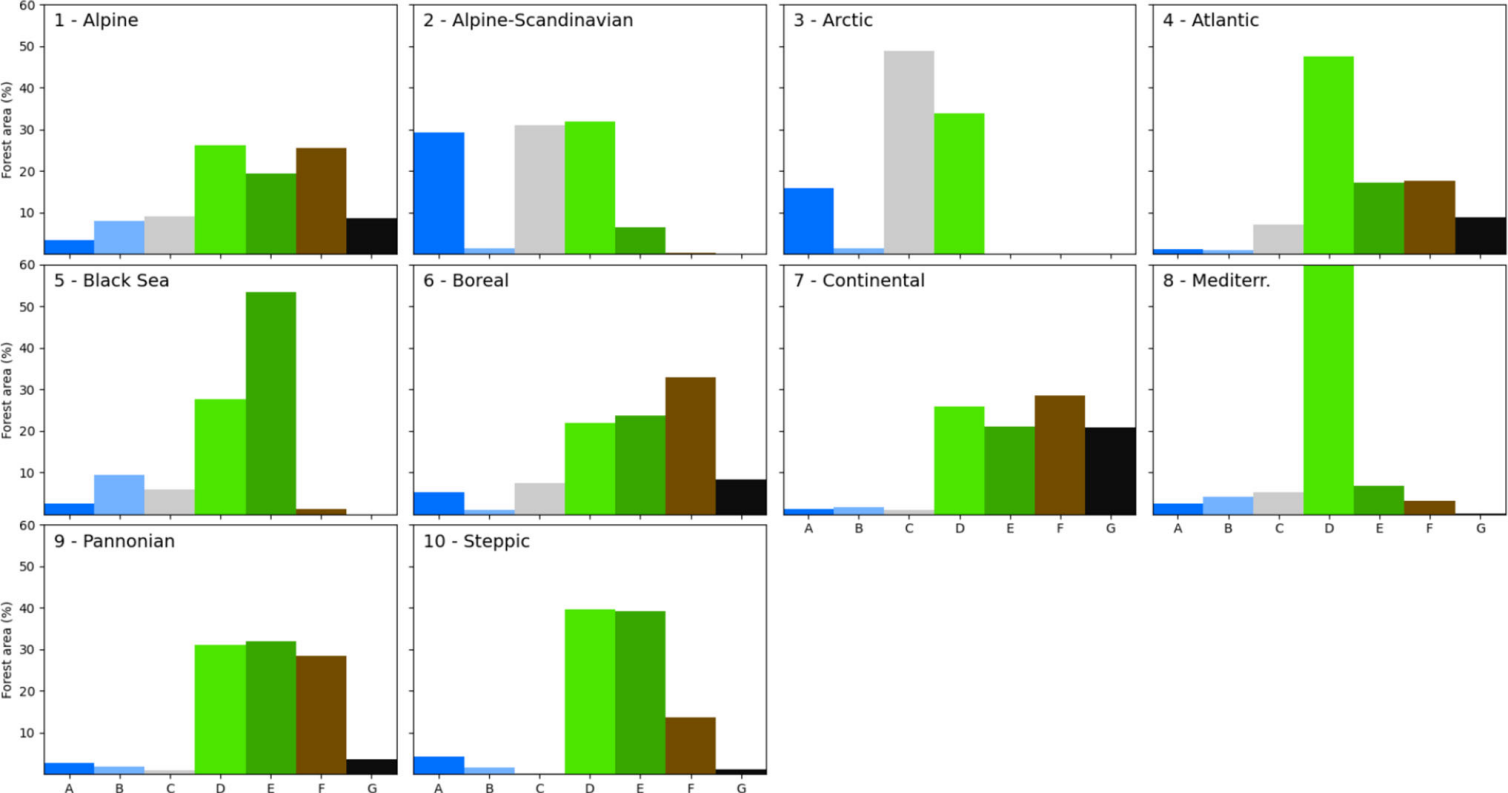
Frequency distribution of forest archetypes in European Biogeographical regions (EEA 2025a) (panels 1–10). Archetypes: **A** Primary forests and strictly protected forests, **B** Other protected forests, **C** Unprotected FNAWS, **D** Low-intensity forest use, **E** Medium-intensity forest use, **F** High-intensity forest use, **G** Very high-intensity forest use. The percentage of forest area for archetype D in the Mediterranean (panel 8), 78%, was truncated for better readability of the charts

Archetypes A and B, which correspond to primary forests and protected forests outside wood production regimens, are marginal, covering a combined extent of less than 7% of the forests within the study area (Fig. 2B). These archetypes are present in the northern regions of Europe, specifically in the Alpine-Scandinavian and Boreal regions, as well as in the Alpine region, particularly in the Carpathians, Alps, Pyrenees, and Rhodopes and Balkan mountains in Bulgaria. They are also found in the Apennines and across the Mediterranean region (Table 3). Archetype A represents a significant proportion of the forests in the Alpine-Scandinavian region, at 29.3% (Fig. 3 and Table S1 in supplementary material).

### Archetype C

Archetype C, representing unprotected FNAWS, barely covers 7% of the forests within the study area. This archetype is generally found in areas of difficult accessibility, such as the steep slopes of high montane ranges in the Alpine and Alpine-Scandinavian regions, particularly in the Alps, Pyrenees, Carpathians, and Scandes (Fig. 2A). It is also found in low productivity areas of the Boreal and Mediterranean regions and in the Atlantic region (Table 3).

### Archetype D

Archetype D, which describes low forest use intensity, is the largest archetype in the study area, accounting for 36% of the forest area (Fig. 2B). Large areas of this archetype are found in the Mediterranean, Boreal, Continental, and Atlantic regions, together covering 88% of the archetype’s extent. This archetype is also found in the Alpine and Alpine-Scandinavian regions (Table 3).

Archetype D is the largest archetype in the Mediterranean and Atlantic regions, comprising 78% and 48% of each regions’ forest area, respectively (Fig. 3). This archetype is also prevalent in the Steppic, Alpine-Scandinavian, and Alpine regions, at 40%, 32%, and 26%, respectively. This includes the montane ranges of the Scandes, Alps, Pyrenees, and southern Carpathians (Fig. 2A). The spatial distribution of this archetype is generally influenced by climatic constraints on tree growth, the remoteness of the forest areas, or a combination of both factors, except in the Atlantic region, where forests in this archetype exhibit a highly fragmented pattern at landscape level.

### Archetype E

Archetype E, which represents medium-intensity forest use, covers 18% of the forests within the study area. Most of this archetype, 69%, is found in the Boreal and Continental regions (Fig. 2A and Table 3). Other significant areas of archetype E occur in the Atlantic region, including the montane ranges of northern Spain, the Alpine region in the Alps, Carpathians, and Rhodopes and Balkan mountains in Bulgaria, and the Mediterranean region (Table 3).

### Archetypes F and G

Areas of high and very high forest use intensity, delineated by archetypes F and G, represent around one-third of the study area (Fig. 2B). Large zones of these archetypes are found in the Boreal and Continental regions, where they cover 77% and 81% of each region, respectively (Table 3 and Table S1 in supplementary material). In addition, these two archetypes cover a significant proportion of forests in the Alpine, Pannonian, and Atlantic regions, with 34%, 32%, and 26%, respectively (Fig. 3). On the map in Fig. 2A, archetypes F and G are also evident in central Portugal, northwestern Spain, southwestern France, several regions of Central Europe, the lower areas of the Carpathians, and southern areas of Sweden, Finland, and Belgium. Factors primarily associated with soils and climate, which drive high plant productivity in these areas, along with forest history and socio-economic features, explain the current distribution of these archetypes.

### Wood production in archetypes D–G

**Fig. 4:**
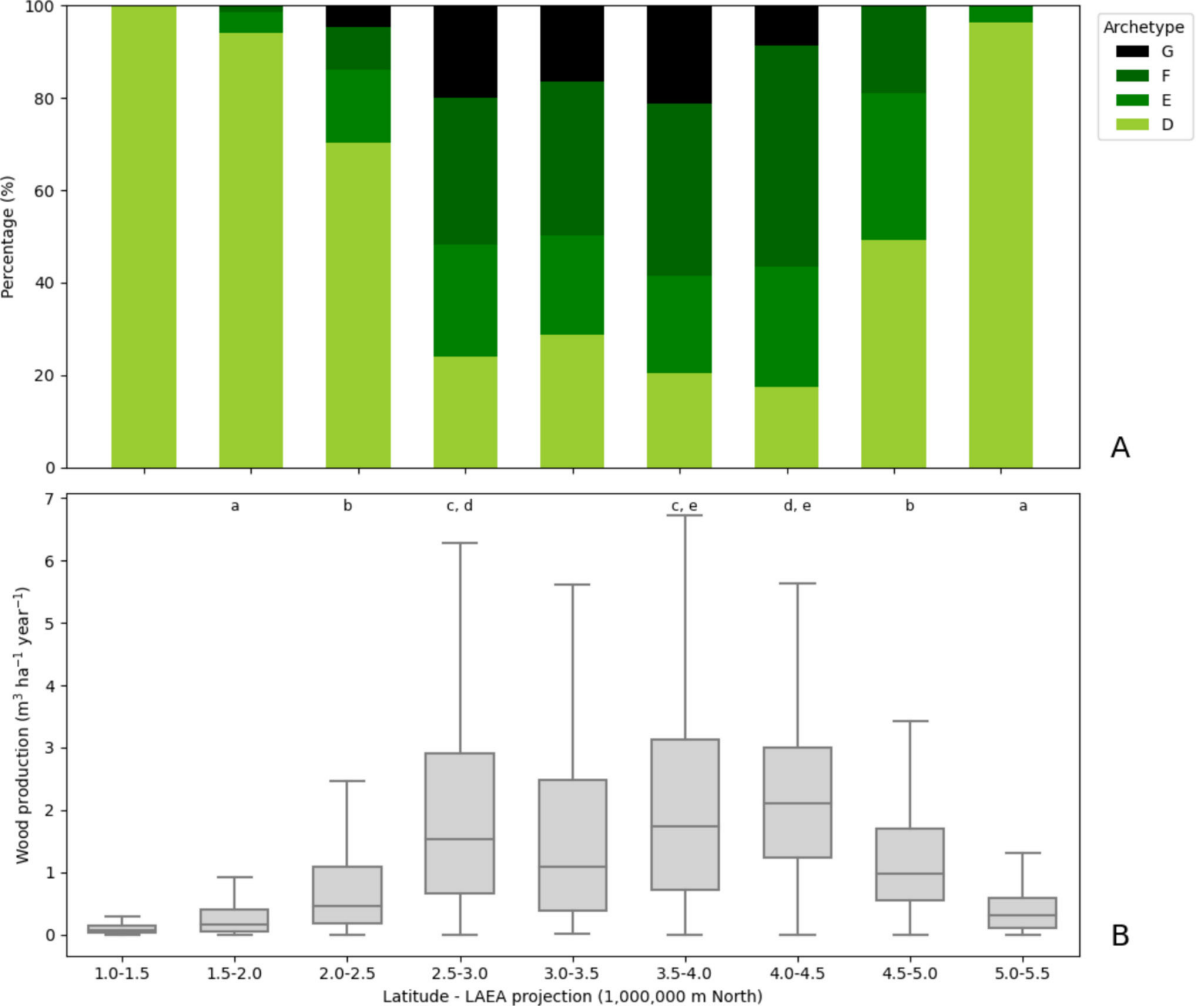
**A** Proportion of forest area covered by archetypes **D**–**G** across nine latitudinal gradients in Europe. **B** Wood production in forest areas within archetypes **D**–**G** across nine latitudinal gradients in Europe. Letters indicate pairs of latitudinal slices where no statistically significant difference between the means of wood production was found at the 95% confidence level, as determined by the Tukey HSD test. Outliers are not shown for better readability of the chart. Archetypes in the legend: **D** Low-intensity forest use, **E** Medium-intensity forest use, **F** High-intensity forest use, and **G** Very high-intensity forest use. LAEA: Lambert Azimuthal Equal Area. The first and last latitudes in the ranges, 1 000 000 m North and 5 500 000 m North, correspond to the geographical coordinates 32.0° N and 72.7° N, respectively. Note that archetypes **A**–**C** are not considered in this Figure

The map in Fig. 2A shows that archetypes D to G, which represent the four wood production archetypes, generally exhibit a latitudinal pattern of wood production. Specifically, low wood production is common in the southern regions, medium to very high in mid-latitudes, and medium to low in the northernmost zones of the continent. This pattern is corroborated by the assessment of archetypes and wood production distribution across latitudinal slices (Fig. 4A, B). The first three slices, at lower latitudes, exhibit lower levels of wood production than the four slices at mid-latitudes (from 2,500,000 m North), with lower wood production again in the last two slices at higher latitudes (from 4,500,000 m North) (Fig. 4B). This is consistent with the proportion of area covered by archetypes describing forest use intensity across the latitudinal slices (Fig. 4A). Specifically, slices with a major proportion of archetype D (low-intensity forest use) are associated with low wood production, while slices with a major proportion of high and very high forest use intensity (archetypes F and G, respectively) are associated with higher levels of wood production. A longitudinal pattern of wood production was not found (not shown).

The one-way ANOVA test revealed a significant difference in mean wood production across the nine latitudinal slices (*p* < 0.001) (Fig. 4B). However, the Tukey HSD test indicated that no significant difference between the means of wood production was found at the 95% confidence level among some pairs of slices, particularly between slices at lower latitudes (the first three slices) and slices at higher latitudes (the last two slices), which exhibited lower wood production than the slices at middle latitudes. Likewise, some slices in the middle latitude region do not show significant differences in wood production among them (Fig. 4). In summary, this assessment revealed significant differences in wood production between the more southern slices and the mid-latitude slices, and between these and the higher latitude slices, which correspond to the landscape patterns described by the archetypes.

The assessment of wood production across archetypes D–G revealed an increasing mean amount of wood production, as shown in Table 4. The one-way ANOVA test indicated a significant difference in mean wood production across the four archetypes (*p* < 0.001). Subsequent analysis using the Tukey HSD test confirmed that all pairwise comparisons between the archetypes showed significant differences in mean wood production (*p* < 0.001). These results suggest that the archetypes exhibit distinct characteristics regarding use intensity and environmental features, which contribute to the observed variations in wood output per unit of time and space.

**Table 4:** Wood production of forest archetypes D–G. There is a significant difference in the mean across the four archetypes and across all pairwise comparisons between archetypes (one-way ANOVA and post hoc Tukey HSD test, both at *p* < 0.001)

Forest archetype	Mean wood production [CI 95%], SD (m^3^ ha^−1^ year^−1^)
D—Low-intensity forest use	0.44 [0.42–0.45], 0.29
E—Medium-intensity forest use	1.48 [1.46–1.50], 0.29
F—High-intensity forest use	2.86 [2.83–2.90], 0.55
G—Very high-intensity forest use	5.57 [5.46–5.67], 1.71

### Mean patch size in forest archetypes

**Fig. 5:**
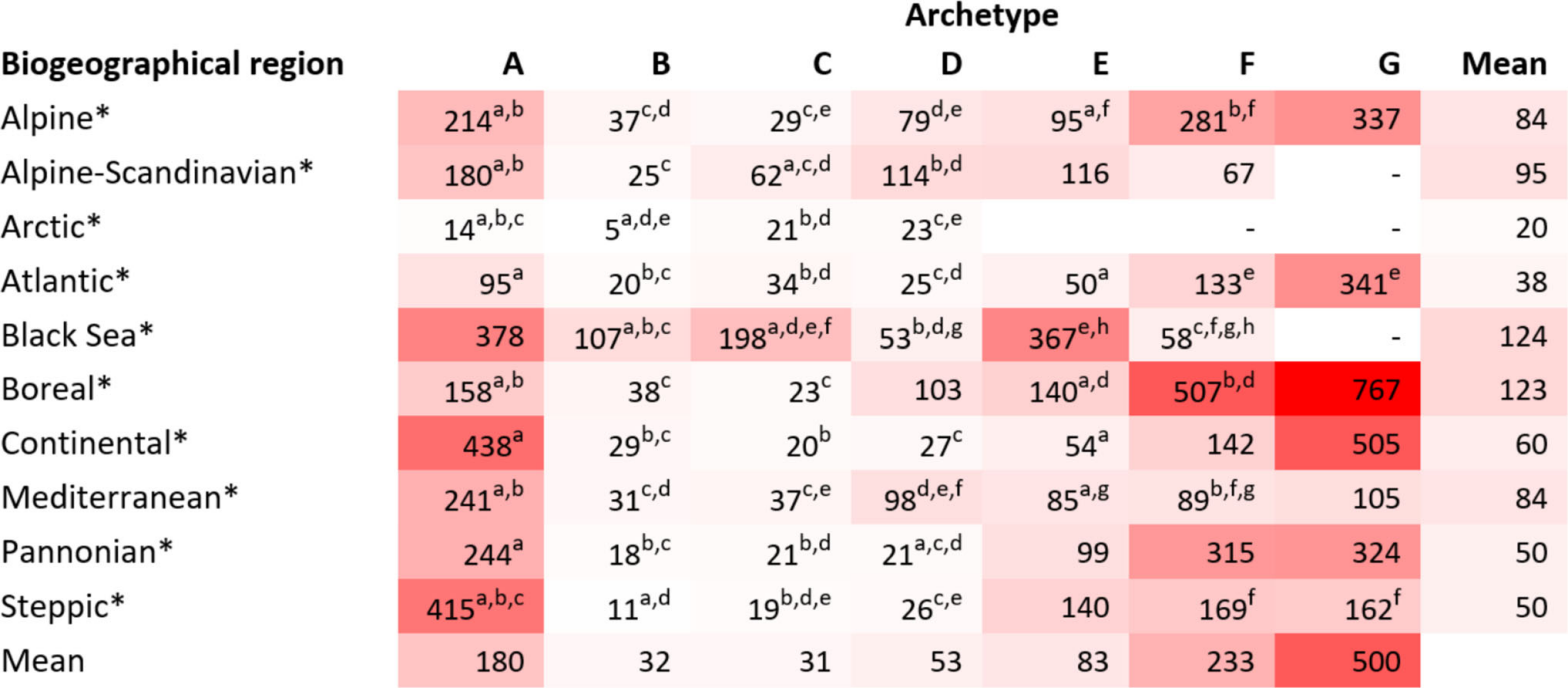
Heatmap showing the mean patch size (MPS) of forests across archetypes and biogeographical regions. Numbers represent hectares. MPSs were calculated in forest patches greater than two hectares. Colours represent a gradient from the lowest value (white) to the highest value (red). Archetypes: **A** Primary forests and strictly protected forests, **B** Other protected forests, **C** Unprotected FNAWS, **D** Low-intensity forest use, **E** Medium-intensity forest use, **F** High-intensity forest use, **G** Very high-intensity forest use. *One-way ANOVA significance: *p* < 0.01. The small letters represent pairs of groups within each biogeographical region for which the Tukey HSD test revealed no significant difference between their means (*p* < 0.05)

The assessment of MPS revealed 2,255,332 patches greater than two hectares across the study area. We found a pronounced difference in MPS among archetypes and biogeographical regions (Fig. 5). The one-way ANOVA test revealed a significant difference (*p* < 0.01) in the logarithm of MPS across archetypes within each biogeographical region. The Tukey HSD tests provided information on pairs of groups (archetypes) within each biogeographical region for which a significant difference in MPS was not found at the 95% confidence level.

Archetype G, characterised by very high-intensity forest use, exhibits the largest MPS across all archetypes in the study area at 500 ha, with peaks in the Boreal and Continental regions at 767 and 505 ha, respectively. Likewise, this archetype generally shows the largest MPS across the largest regions, except in the Mediterranean, where archetype A exhibits the larger MPS at 241 ha. In contrast, archetypes B, C, and D, corresponding to other protected areas, unprotected FNAWS, and low-intensity forest use, exhibit the lowest MPS across all the archetypes in Europe, at 32, 31, and 53 ha, respectively, suggesting more fragmented forests. Interestingly, we found an increasing gradient of MPS from archetypes B and C to archetype G in Europe (bottom row in Fig. 5). In other words, more intense forest use appears to be associated with larger MPS across these archetypes. Despite the lack of significance in the Tukey HSD test for several pairs of groups, this pattern generally holds true across biogeographical regions (Fig. 5).

The archetypes located at both extremes of the archetypes gradient—A on the left side, and F and G on the right side of Fig. 5—account for the largest MPS across Europe. This information may yield important insights for forest protection and restoration. For example, the low MPS observed in archetype C, unprotected FNAWS, across regions, averaging 31 ha, may pose constraints for the creation of new protected areas and the implementation of restoration measures in forests within this archetype, a point to which we shall return later in the discussion section.

### Results at country level

**Fig. 6:**
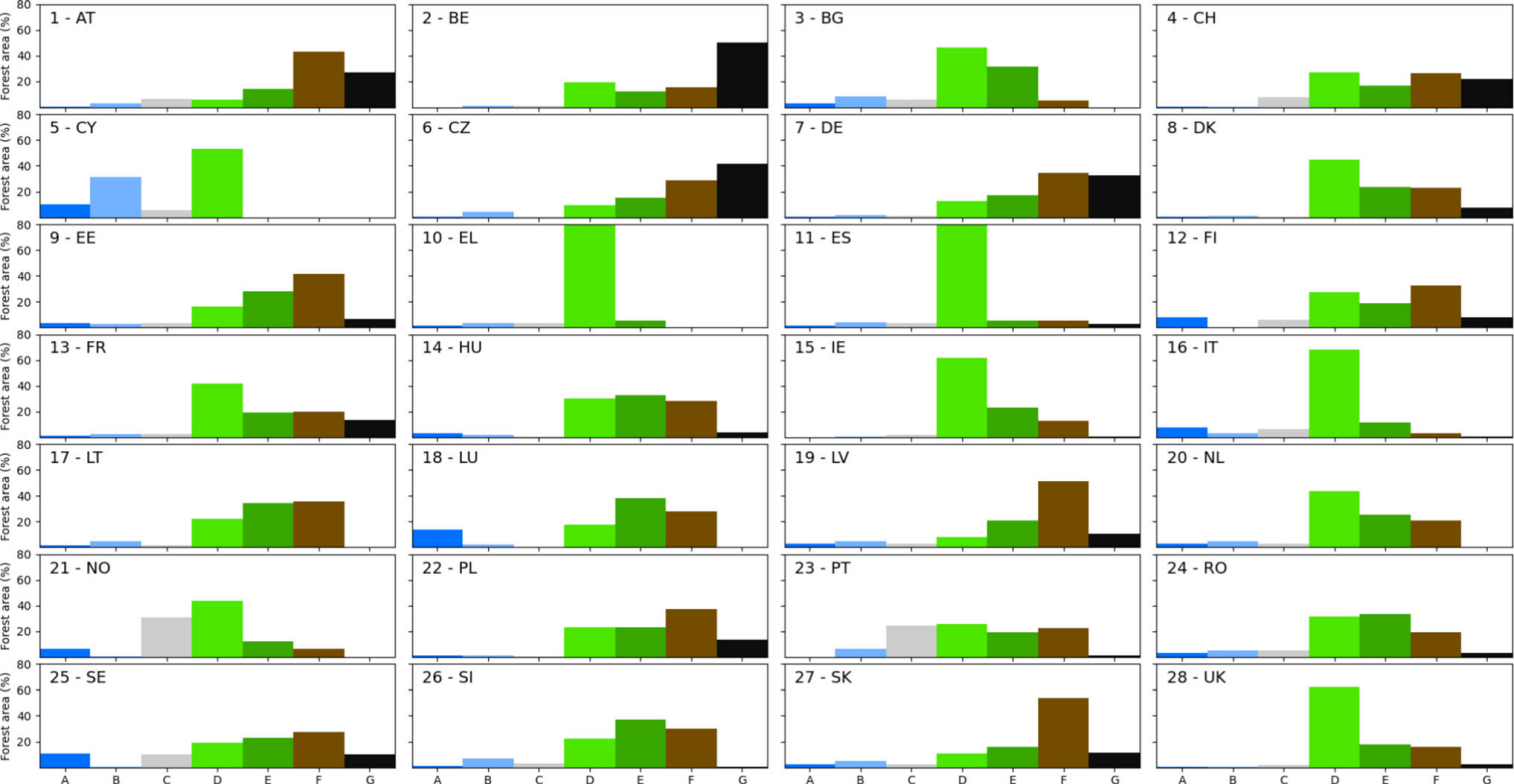
Frequency distribution of forest archetypes in European countries (panels 1–28). Archetypes: **A** Primary forests and strictly protected forests, **B** Other protected forests, **C** Unprotected FNAWS, **D** Low-intensity forest use, **E** Medium-intensity forest use, **F** High-intensity forest use, **G** Very high-intensity forest use. The percentage of forest area for archetype D in Greece (panel 10), 86.1%, was truncated for better readability of the charts

The results on the frequency distribution of archetypes per country are shown in Fig. 6 and in Table S2. The distribution of archetypes provides information on the characteristics of forests in each country. As shown in Fig. 2A, archetype D, representing low forest use intensity, is the predominant archetype in southern European countries, particularly in Spain, Italy, Greece, Cyprus, and Bulgaria, covering more than 46% of the forest area in each (Fig. 6, panels 3, 5, 10, 11, and 16). Similarly, this archetype is predominant in the UK and Ireland, accounting for 62% in each (Fig. 6, panels 15 and 28). However, in these two countries, there are a higher proportion of archetypes E and F, representing medium- and high-intensity forest use, compared to the southern countries, indicating a generally greater intensity of forest use. France and the Netherlands exhibit a similar pattern regarding the predominance of archetype D, with 42% and 43%, respectively. However, they have an even greater proportion of medium to high forest use intensity archetypes, as well as a very high-intensity forest use archetype in France.

With a few exceptions, the primary forests and nature conservation forest archetypes, A and B, are marginal across countries, with extents generally well below 10%. Similarly, archetype C, which describes unprotected forests not used for wood production, shows a rather limited extent in most countries, with the exceptions of Norway, Portugal, and Sweden, where it represents 31%, 25%, and 10%, respectively.

Central European countries exhibit a pronounced proportion of forest archetypes characterised by medium to high forest use intensity. This is evident in Austria, Switzerland, Czechia, Germany, Hungary, Luxembourg, Poland, Slovenia, and Slovakia, as well as in Romania in Eastern Europe (Fig. 6, panels 1, 4, 6, 7, 14, 18, 22, 24, 26, and 27). A similar pattern of medium to high forest use intensity is present in the Nordic countries, namely Denmark, Sweden, and Finland, and in the three Baltic Republics: Estonia, Latvia, and Lithuania (Fig. 6, panels 8, 9, 12, 17, 19, and 25). In addition to historical legacies of intense forest use in Central Europe (Kaplan et al. 2009; McGrath et al. 2015), favourable climatic factors and socioeconomic drivers shape the current pattern of forest use intensity archetypes in this region.

Five countries exhibit more than 20% of their forest area in archetype F, which describes very high forest use intensity: Belgium, Czechia, Germany, Austria, and Switzerland (Fig. 6, panels 1, 2, 4, 6, and 7). Malta shows no wood production and has almost no forest area; therefore, it is not included in Fig. 6.

### Sensitivity analysis and benchmarking

Results of the sensitivity analysis and the benchmarking are shown in the supplementary material S1.

## Discussion

This study provides a novel map of forest archetypes, enhancing the knowledge base of European forests. In addition to the spatial delineation of the seven archetypes, the most salient results indicate that around one-third of European forests experience high and very high levels of forest use intensity. This proportion is even higher in 13 out of 29 countries. Meanwhile, the forest area devoted to nature conservation and passive use, and excluded from wood production, comprises about 13% of the total forest area, which falls short of the 60% of unmanaged forest necessary for achieving high biodiversity levels in many jurisdictions (Duflot et al. 2025) (Fig. 6).

We found that 50% of European forests fall within medium to very high wood production regimens, represented by archetypes E, F, and G. Combined with archetype D—Low-intensity forest use, these account for 87% of forests potentially under wood production regimens. This is not surprising, considering that many protected areas, including Natura 2000 sites, which represent 23% of the EU forest area (Maes et al. 2020), do not restrict forestry activity (MCPFE 2003; European Commission 2015). Forestry activity is restricted only in strictly protected areas, i.e. IUCN’s protected area categories Ia, Ib, and II (Dudley and Phillips 2006). Even if in category II, minimal interventions may be allowed (MCPFE 2003).

One salient finding from the perspective of nature and biodiversity conservation is the limited extent of archetype A—Primary forests and strictly protected forests, which represent only 4.4% of European forests. Notably, in the EU, a large proportion of mapped primary forests, 87%, fall within strictly protected areas (Barredo et al. 2021). These figures align with the 3.6% of strictly protected forests reported by Nagel et al. (2025) using national-level data in Europe. Nevertheless, we emphasise that the extent of the mapped (known) primary forests most likely falls short of the potential extent of these forests due to gaps in mapping across Europe (Sabatini et al. 2020; Barredo et al. 2025). This implies that the extent of archetype A in our map might be underestimated to some degree.

Similarly, the proportion of other protected forests within FNAWS, which are excluded from wood production regimens and described by archetype B, is marginal at 2.3%, with the lowest MPS across archetypes at 14 ha, indicating highly fragmented forests. This indicates that the proportion of protected forests legally excluded from wood production is around  7% of the total European forest extent. We note that protected forests falling within FAWS were classified in archetypes D to G according to the wood production level (Table 2).

This situation is exacerbated in 17 out of 29 countries, namely, Austria, Belgium, Czechia, Denmark, Estonia, France, Germany, Greece, Hungary, Ireland, Lithuania, Poland, Portugal, Slovakia, Spain, Switzerland, and the UK, where the combined proportion of archetypes A and B is less than 7%. Furthermore, in 23 countries, the proportion is below 10%, including Finland, Latvia, the Netherlands, Norway, Romania, and Slovenia, in addition to those mentioned above.

Unprotected forests in FNAWS, corresponding to Archetype C, cover around 7% of European forests. These forests represent potential options for fulfilling the aims of the EU Biodiversity Strategy, which seeks to protect at least 30% of EU land, of which 10% strictly protected (European Commission 2020). However, assessing the ecological value and condition of forests in archetype C is a prerequisite for adopting protection measures. This involves evaluating the biodiversity, condition, and ecosystem services values on each patch, as well as their extent and landscape features. Notably, forests in this archetype exhibit one of the lowest MPS across biogeographical regions, ranging from 19 to 62 ha, except in the Black Sea region, where they reach 198 ha.

Forests under low-intensity use, represented by archetype D, constitute the largest archetype in Europe, covering 36% of the forest area. These forests may be subject to low-intensity management or could currently be unmanaged or abandoned. Thus, while they are classified as FAWS, in some regions, they may exhibit little to no wood production, i.e. below 0.1 m^3^ ha^−1^ yr^−1^. Forests in this archetype are often located in regions with climatic limitations for tree growth, such as the dry areas of the Iberian Peninsula and other Mediterranean regions, where this archetype is prominent (Fig. 3, panel 8), the coldest zones of the Northern Boreal, and areas in high mountains (Fig. 3, panels 1 and 2).

Despite assessing forest fragmentation is not within the aims of this study and more fragmentation metrics, such as the distance among patches, are necessary for more robust conclusions, the information on MPS across archetypes and regions raises several questions that warrant further research. The fact that natural forests in archetype A and the most intensively used forests in archetypes F and G exhibit the largest MPS across Europe suggests that well-conserved forests, on one hand, and forest under very intensive forestry, on the other, experience the lowest levels of forest fragmentation. In contrast, forests with less stringent protection levels (archetype B), un-protected forests in FNAWS (archetype C), and forests where productivity is low (archetype D) are generally more fragmented exhibiting smaller MPS, which may pose a challenge for nature conservation measures. However, these forests with lower MPS could be as important as those with larger MPS from a biodiversity conservation point of view (Fahrig 2020). These findings emphasise the importance of policy measures oriented towards connectivity-focused conservation and restoration strategies, such as the targets on forest connectivity adopted in the EU Regulation on Nature Restoration (European Union 2024), which should contribute to mitigating the issue of the high proportion of small forest patches and of the small proportion of large strictly protected areas in Europe found in Nagel et al. (2025).

While archetypical patterns of land systems have been frequently addressed (Václavík et al. 2013; van der Zanden et al. 2016; Levers et al. 2018; Dou et al. 2021), to our knowledge, no map of forest archetypes existed in Europe before this study. The results of this study help fill this gap. The archetypes map provides synthetic information that facilitates the understanding of the extent and distribution archetypal forests, which describe levels of forest use intensity and natural features at the landscape level across Europe. It helps to account for how human modification and forest use impact European forests. Additionally, the map aids in understanding the spatial distribution and extent of natural forest, protected and unprotected forests outside wood supply regimes, as well as forests under various levels of wood supply. We caution that the archetypes map describes patterns distinguishable at large scale. Further examinations at local scales would require complementary fine-grained data because large-scale patterns may not always hold at such local scales (Levers et al. 2018; Eisenack et al. 2021).

Our study aligns with several research efforts that have advanced knowledge on the spatially explicit representation of landscape patterns resulting from the interactions between human activities and the environment. Notably, these include studies developing archetype maps and trajectories of land systems at continental level in Europe (van Eupen et al. 2012; van der Zanden et al. 2016; Levers et al. 2018; Dou et al. 2021), global land system archetypes (Ellis and Ramankutty 2008; van Asselen and Verburg 2012; Václavík et al. 2013), as well as regional assessments in West Africa (Wingate et al. 2023), and the European Mediterranean domain (Malek and Verburg 2017).

Despite significant differences in aims, data, scales, and assessed features, a comparison of our map with previous maps describing forest management approaches by Hengeveld et al. (2012), Nabuurs et al. (2019), and more recently Scherpenhuijzen et al. (2025), suggests an overall good fit between the archetypes and the levels of management intensity. This is evidenced, for instance, in areas of high forest use intensity, such as southern Sweden, southern Finland, central Portugal, the Czech Republic, and southwestern France. Beyond differences in the scope of the studies and the baseline data employed, the previous maps focus in forest management categories, including silvicultural descriptors mapped often at the stand level. Examples include cleared forest areas and the presence of commercial tree species used by Scherpenhuijzen et al. (2025). In contrast, our map represents archetypes at the landscape level, mapping large patterns and delineating forest use intensity rather than forest management regimens, which are often described at stand level. These aspects distinguish our study from previous studies focusing on forest management regimes.

Another remarkable difference and novelty of our map is the emphasis on the description of protected, strictly protected, and primary forests, as well as unprotected forest outside wood production regimens. In this respect, Scherpenhuijzen et al. (2025) appear to underestimate the area of unmanaged forests, at 0.5% of European forests, while the present study found a proportion of 4.4% for strictly protected and primary forests, 2.3% for other protected forests outside FAWS, and 6.7% for unprotected forests outside FAWS. This in agreement with the 3.6% found by Nagel et al. (2025) for strictly protected areas alone, taking into consideration some differences in the countries covered in both studies. Nevertheless, our map also features a quantitatively based gradient of forests under wood production regimes across four archetypes, generally associated with increasingly intensive forest management approaches (Duncker et al. 2012a; Barredo et al. 2024). Consequently, the representation of forest archetypes in our map provides crucial information for biodiversity and nature conservation, which, to the best of the authors’ knowledge, was not previously available in a harmonised and spatially explicit seamless manner.

### Limitations and uncertainties

We identified some aspects that suggest limitations in this study. The first relates to uncertainties and potential inaccuracies in the data we used. Although the data are reliable, validated, and sourced from peer-reviewed studies, we acknowledge that these uncertainties could have propagated into the forest archetypes map presented here. For instance, the map of wood production involves, among other methodological steps, the downscaling of statistics from administrative units to 1-km grid cells, which may result in greater uncertainty compared to the primary forest and protected area data sets, which are derived from field surveys and delineation approaches. Another issue could be the difficulty in applying the definition of FAWS in situations like voluntarily set-aside forests in Sweden, which can also include productive forests (> 1 m^3^ ha^−1^ yr^−1^). In addition, the coordinates of these forests are not publicly available; therefore, they were classified like any other forest in the FAWS map unless there are other limitations in place (Avitabile et al. 2024). Second, the expert-based forest archetype classification system used in our study provides a useful and transparent approach for systematically addressing the spectrum of archetypes present in European forests. However, we acknowledge that any classification system inherently involves a degree of arbitrariness necessary for category separation. While automated methods can serve as an alternative to expert-based methods for land archetype mapping, van der Zanden et al. (2016) compared the two approaches and concluded that there is significant agreement between them. Nonetheless, some classes that are important for landscape function from an expert-based perspective may not be sufficiently differentiated in automated methods. Furthermore, due to its transparent nature, expert-based approaches are easy to replicate when newly updated data become available for future assessments.

Third, a limitation of archetype mapping, in general, is the lack of readily available validation data. This is evidenced by the absence of traditional accuracy-oriented validation practices in previous land archetype mapping studies (e.g. Václavík et al. 2013; van der Zanden et al. 2016; Levers et al. 2018). One of the challenges in assessing the performance of archetype mapping is the inherently landscape-level nature of the archetypes themselves. While archetype maps describe landscape pattern characteristics, traditional accuracy-oriented validation focuses on local data, which restricts its applicability in this context (Dou et al. 2021). The local nature of validation data does not always adequately capture the landscape pattern characteristics of the archetypes, an aspect that warrants further research. In light of this limitation, we conducted firstly, a sensitivity analysis to determine the extent to which the selection of thresholds for wood production influences the archetype classification results. The analysis revealed only minor differences in most cases. Secondly, a benchmarking between the results of this study and the areal accounts of Nagel et al. (2025) resulted in an overall agreement of 87%, with agreement per biome of 97.1% in the Mediterranean, 99.1% in the Temperate, and 70.2% in the Boreal.

An additional limitation is that other European areas of high biodiversity value, such as the Dinaric Alps in the Balkan Peninsula or the Ukrainian part of the Carpathians, which are exposed to significant anthropogenic pressures, are not included in the archetypes map due to spatial gaps in the underlying data sets used in this study.

## Conclusion and Policy implications

This study provides a nuanced representation of European forests by considering whether forests are available for wood supply or excluded from forestry activities. The results of this study could be relevant for policy assessment and implementation, such as the land protection targets specified in the EU Biodiversity Strategy to 2030 (European Commission 2020) and the restoration targets proposed in the EU Regulation on Nature Restoration (European Union 2024). In the first case, targets for protecting at least 30% of the EU land area, with one-third of that area strictly protected, can be assessed in relation to forests by considering the proportion of archetypes in different regions and countries, among other factors. This can be achieved, for instance, by maximising the protection of valuable forests located outside wood production regimens or under low forest use intensity. In particular, the focus should be on the strict protection of non-protected primary forests in Archetype A, enhancing the protection of forests in Archetype B to more stringent levels, considering important forests for biodiversity in Archetype C to be included under protection regimes, and identifying valuable areas in Archetype D as candidates for protection. These actions should consider the delineation of protected buffer areas around primary and strictly protected forests in archetype A (Barredo et al. 2021). Which will support forest restoration in a well-connected landscape representing a win–win pathway for biodiversity conservation and climate change mitigation.

In the second case, answering questions such as what type of forest should be restored and where is crucial. These questions can be informed by assessing the forest archetypes map alongside other data, such as forest condition and the private or public nature of the forest land. Additionally, information on the spatial distribution and extent of archetypes at the country level offers insights into the shift towards a closer-to-nature management approach in the EU (European Commission 2023b). Understanding where and how much an archetype is present in a region or country is crucial for a complete understanding of the current state of European forests, particularly in synergy with novel studies assessing forest condition (e.g. Maes et al. 2023).

While the protection designation in IUCN’s protected area categories III–VI, as well as in Natura 2000 sites in the EU, permits silvicultural operations within certain limits and generally yields positive results for biodiversity conservation (e.g. Ceccherini et al. 2023), it can also lead to undesired outcomes when law enforcement is not adequately applied. This has been observed in Romania, Poland, Estonia, and Slovakia where logging operations in Natura 2000 sites have resulted in infringement cases INFR(2020)2033, INFR(2016)2072, and INFR(2021)4029, INFR(2018)4076, respectively (European Commission 2025). At this juncture, the aim of the EU Biodiversity Strategy to 2030, which seeks to strictly protect 10% of land and ensure the strict protection of the last remnants of primary and old-growth forests in the EU (European Commission 2023a), represented in archetype A, are pressing priorities for the conservation of valuable forests before it is too late (Mikolāš et al. 2023; Barredo et al. 2025).

Land is a finite resource and forms the basis for life on Earth. Well-balanced land management that sustains the provision of ecosystem services while maintaining ecosystems in good condition is fundamental for the bioeconomy (European Commission 2018). The intensity of forest use is significant in European forests, as shown in the archetypes map. This aligns with evidence indicating an increased use of wood across Europe (Senf et al. 2018; Ceccherini et al. 2020; Senf and Seidl 2021), which affects other services, such as the forest carbon sink, thereby impacting climate regulation services (Korosuo et al. 2023). The planetary boundaries lie well before the point of ecosystem degradation (Steffen et al. 2015), as restoration at that stage is costlier than conservation and the damage is often irreversible.

## Supplementary Information

Below is the link to the electronic supplementary material.

Supplementary file1 (PDF 1088 KB)
