# Supplementary material for: Mapping European forest archetypes
Source: Ambio. 2025 Dec 3;55(7):1619–38. doi: 10.1007/s13280-025-02318-2 (PMC13230407; doi:10.1007/s13280-025-02318-2)
Supplement: Supplementary file 1 — Supplementary file1 (PDF 1088 KB) [file 13280_2025_2318_MOESM1_ESM.pdf]

Ambio

Supplementary Information - Appendix

This supplementary information has not been peer reviewed.

Title: **Mapping European forest archetypes**

Table S1. Percentage of forest area by archetype per biogeographical region in Europe. Archetypes: Archetypes: A) Primary forests and strictly protected forests, B) Other protected forests, C) Unprotected FNAWS, D) Low intensity forest use, E) Medium intensity forest use, F) High intensity forest use, G) Very high intensity forest use. Croatia is not included in this assessment due to a lack of data on wood production. Data for Malta is not considered because it shows no wood production and almost no forest area. Note rounding effect in the percentages.

		**Proportion of archetype's forest area (%)**						
**Biogeographical region**	**Forest area (1000 km^2)**	**A**	**B**	**C**	**D**	**E**	**F**	**G**
Alpine	162	3.2	8	9.1	26.2	19.4	25.6	8.5
Alpine-Scandinavian	85	29.3	1.3	31	31.8	6.4	0.2	0
Arctic	0.1	15.8	1.3	48.9	34	0	0	0
Atlantic	182	1.2	0.9	7	47.5	17.1	17.6	8.7
Black Sea	3	2.5	9.4	5.9	27.6	53.3	1.3	0
Boreal	579	5.2	0.9	7.3	21.9	23.6	32.9	8.3
Continental	391	1.2	1.7	1.1	25.8	21.1	28.4	20.7
Mediterranean	303	2.5	4	5.2	78	6.7	3.3	0.2
Pannonian	25	2.7	1.7	0.9	31	31.9	28.3	3.5
Steppic	2	4.1	1.6	0.3	39.7	39.2	13.7	1.2
Total	1,733	4.4	2.3	6.7	36.3	18.4	22.7	9.2

Table S2. Percentage of forest area by archetype per country. Archetypes: Archetypes: A) Primary forests and strictly protected forests, B) Other protected forests, C) Unprotected FNAWS, D) Low intensity forest use, E) Medium intensity forest use, F) High intensity forest use, G) Very high intensity forest use. Croatia is not included in this assessment due to a lack of data on wood production. Data for Malta is not considered because it shows no wood production and almost no forest area. Note rounding effect in the percentages.

		Proportion of archetype's forest area (%)						
**Country**	**Forest area (km^2)**	**A**	**B**	**C**	**D**	**E**	**F**	**G**
Austria	39,005	0.7	3.3	6.1	5.7	14.1	43.1	27.1
Belgium	6,901	0	1.4	1.2	19.3	11.9	15.7	50.4
Bulgaria	38,941	3.2	8.4	5.9	46.4	31.3	4.8	0
Cyprus	1,708	10.1	31.4	5.4	53	0	0	0
Czech Republic	27,889	0.5	3.9	0.6	9.4	15.2	28.6	41.6
Denmark	6,294	0.1	1	0.3	44.9	23.7	22.7	7.3
Estonia	24,418	3.2	2.4	3.1	16	27.5	41.4	6.5
Finland	224,018	7.7	0.4	5.7	27.1	18.7	32.6	7.8
France	172,489	1	2.7	2.2	42	19.1	19.6	13.3
Germany	106,086	0.5	1.6	0.8	12.8	17.1	34.7	32.6
Greece	38,812	1.1	3.6	3.4	86.1	5.5	0.3	0
Hungary	21,466	3	1.8	0.6	30.4	32.6	28.2	3.4
Ireland	7,806	0	0.4	1.5	61.5	23.2	13	0.4
Italy	95,531	7.8	3.2	6	68.1	11.4	3.3	0.2
Latvia	34,133	2.5	4.8	2.5	7.7	20.9	51	10.6
Lithuania	22,021	1.7	4.6	1.7	22	34.4	35.4	0.1
Luxembourg	888	13.6	2.1	0.1	17.9	38	27.8	0.4
Netherlands	3,695	2.9	4.7	2.7	43.3	25.6	20.8	0
Norway	121,812	6.4	0.7	30.6	43.8	12.4	6.1	0
Poland	94,774	1.2	1.1	0.5	23.2	23.1	37.5	13.5
Portugal	32,403	0	6.6	24.7	25.8	19.4	22.2	1.3
Romania	69,211	2.9	4.8	4.8	31.7	33.6	19.3	2.9
Slovakia	21,881	2	4.7	2.1	10.4	15.9	53.7	11
Slovenia	12,374	0.8	6.8	2.7	22.4	37.1	29.7	0.6
Spain	184,265	1.5	3.9	3	79.2	5	5.1	2.4
Sweden	279,966	10.4	0.5	10	18.8	23.2	27.4	9.7
Switzerland	12,288	0.4	0.4	7.8	27	16.5	26.2	21.7
United Kingdom	31,698	0.2	0.5	1.6	62.1	17.6	15.7	2.3
Total	1,732,774	4.4	2.3	6.7	36.3	18.4	22.7	9.2

Sensitivity Analysis

While we used information from previous peer-reviewed studies and ancillary statistics to set thresholds for wood production, these thresholds impact the extent of the archetypes defined based on wood production, namely D, E, F, and G. We conducted a sensitivity analysis to determine how reasonable changes in these wood production thresholds might affect the extent of these four archetypes. To achieve this, we systematically introduced 10 variations in the adopted thresholds of wood production (Table 2 in the manuscript), except for the lower and upper thresholds or archetype D and F, respectively, as they represent the minimum and maximum values of wood production across

the studied area. The variations ranged from -0.5 m^3 ha^{-1} yr^{-1} to 0.5 m^3 ha^{-1} yr^{-1}, in intervals of 0.1 m^3 ha^{-1} yr^{-1}. For example, the wood production range of archetype E, i.e., 1−2 m^3 ha^{-1} yr^{-1}, would result in a range of 0.7−1.7 m^3 ha^{-1} yr^{-1} under a -0.3 m^3 ha^{-1} yr^{-1} variation. We then assessed the changes in the extent of the four archetypes for each introduced variation relative to the adopted thresholds at both European and country levels.

The sensitivity analysis indicates the extent to which changes in the selection of thresholds influence the results of the archetypes' classification. The analysis revealed directly proportional changes in the extent of the archetypes D, E, F, and G as the threshold variations increased in either direction (negative or positive), both at the European and country levels (Figures S1 and S2, respectively). For the whole of Europe, as expected, increases in the thresholds resulted in a simultaneous expansion of archetype D and reduction of archetype G. Conversely, lowering the thresholds reduced the extent of archetype D while expanding that of archetype G. Generally, the archetypes resulting from the introduced changes in the sensitivity analysis maintained their size ranking, except in the more substantial variations below -3 m^3 ha^{-1} yr^{-1}, where a reordering was observed.

At the country level, the effects of threshold variations exhibited distinct patterns depending on regional wood production characteristics (Figure S2). Some countries, such as Hungary, showed greater sensitivity to threshold changes, indicating a significant reclassification of forest areas among the archetypes. In contrast, other regions, such as the Czech Republic and Norway, exhibited smaller changes, suggesting lesser sensitivity to changes in thresholds. Overall, the analysis underscores that smaller variations in the thresholds do not lead to significant changes in the extent of the archetypes, resulting in only minor differences. Conversely, larger variations in the thresholds might significantly influence the classification of forest archetypes.

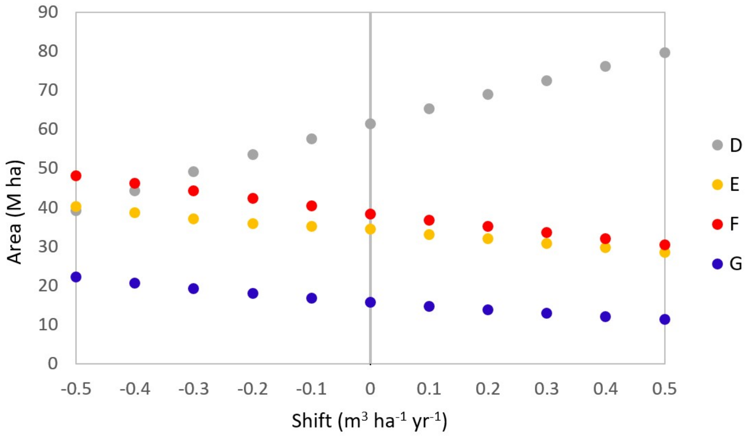

Figure S1. Sensitivity Analysis of forest area of archetypes D, E, F, and G under a range of threshold variations in Europe. Archetypes: D) Low intensity forest use, E) Medium intensity forest use, F) High intensity forest use, G) Very high intensity forest use. The vertical grey line represent the thresholds adopted in the study.

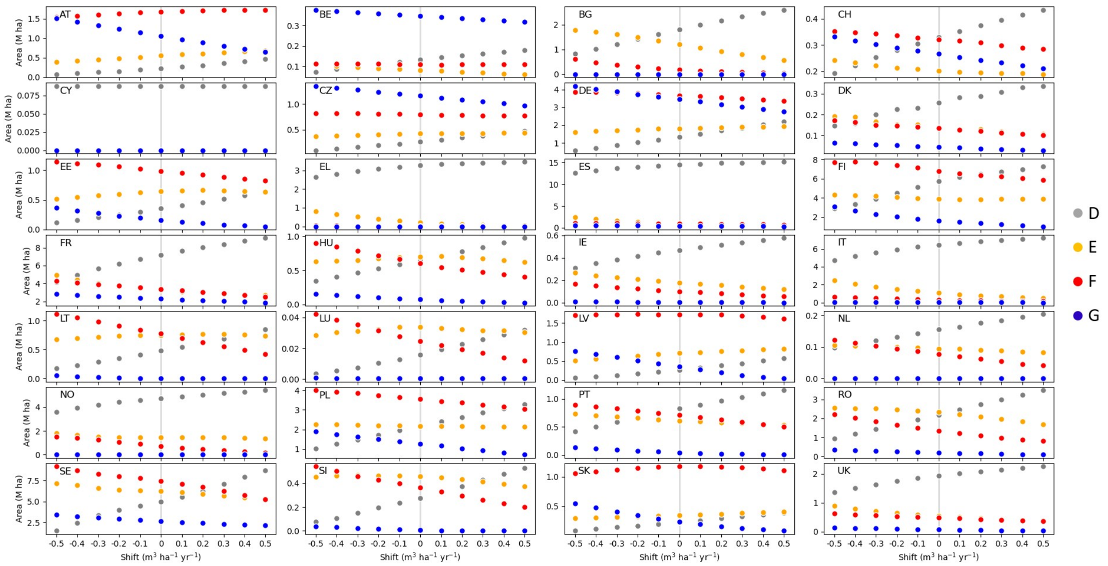

Figure S2. Sensitivity Analysis of forest area of archetypes D, E, F, and G under a range of threshold variations in European countries. Archetypes: D) Low intensity forest use, E) Medium intensity forest use, F) High intensity forest use, G) Very high intensity forest use. The vertical grey lines represent the thresholds adopted in the study.

Benchmarking

We conducted a comparison of the area accounted for by the archetypes in this study versus the area accounted for in Nagel et al. (2025). They compiled data on extensive and intensive forest management across a group of 26 European countries. In addition, they created a database of strict forest reserves for area accounting purposes. As a first step, we selected common countries assessed in both studies, resulting in 22 countries. Second, we classified the countries into three biomes as follows. Mediterranean: Greece, Italy, and Spain; Temperate: Austria, Bulgaria, Czech Republic, Denmark, France, Germany, Hungary, Netherlands, Poland, Romania, Slovakia, Slovenia, and Switzerland; and Boreal: Estonia, Finland, Latvia, Lithuania, Norway, and Sweden. Third, we assumed the correspondence of archetypes in our study with the categories in Nagel et al. (2025). Specifically, Archetype A—Primary forests and strictly protected forests, corresponds to the category of strict forest reserves; Archetype D—Low intensity forest use, corresponds to the category of extensive forest management; while Archetypes E, F, and G, ranging from medium to very high forest use, correspond to intensive forest management. Archetypes B and C were excluded due to a lack of correspondence with categories in Nagel et al. (2025). Although perfect correspondence is not expected due to the significant differences between the two approaches, we calculated the area in agreement per biome and category, as well as the overall agreement in square kilometres and as a percentage of the total forest area. The same approach was applied to the total of all countries. For example, in the Mediterranean biome, Nagel et al. (2025) identified 6929 km² of strict forest reserves, while this study identified 11,572 km² within Archetype A. Therefore, in this case, the agreement is 6929 km², while the disagreement is 4643 km², which is the absolute difference between 11,572 km² and 6929 km².

Results of the comparison indicate a good correspondence between the accounting of both studies (Table S3). The overall agreement is 87% across the three biomes. We found that in the Mediterranean and temperate biomes, the agreement is very good at 97.1% and 99.1%, respectively. However, in the Boreal biome, the agreement is lower at 70.2%. The reason for the lower agreement in the Boreal biome is that Nagel et al. (2025) identified a smaller proportion of extensive forest management compared to what this study found in Archetype D, specifically 2.6% versus to 30.2%, respectively. Nevertheless, considering the major differences in the scope, assumptions, and the baseline data used

in both studies, the benchmarking indicates a good agreement between the area accounts of Nagel et al. (2025) and this study.

Table S3. Comparison of the area allocated to strict forest reserve, extensive forest management, and intensive forest management between this study and Nagel et al. (2025), overall agreement and total area.

Biome	Study	Strict forest reserve		Extensive forest management		Intensive forest management		Overall agreement (km^2)	Total area (km^2)	Overall agreement (%)
		Area (km^2)	%	Area (km^2)	%	Area (km^2)	%			
Mediterranean	Nagel et al.	6,929	2.2	259,783	81.5	51,896	16.3	309,478	318,609	97.1
	This study	11,572	3.6	264,272	82.9	42,765	13.4			
Temperate	Nagel et al.	10,628	1.7	187,280	29.9	428,485	68.4	620,911	626,393	99.1
	This study	9,063	1.4	183,363	29.3	433,967	69.3			
Boreal	Nagel et al.	48,631	6.9	18,435	2.6	639,303	90.5	495,835	706,369	70.2
	This study	64,326	9.1	213,273	30.2	428,770	60.7			
Total	Nagel et al.	66,188	4.0	465,498	28.2	1,119,684	67.8	1,437,188	1,651,371	87.0
	This study	84,961	5.1	660,908	40.0	905,501	54.8			

References

Nagel, T. A., Rodríguez-Recio, M., Aakala, T., Angelstam, P., Avdagić, A., Borowski, Z., Bravo-Oviedo, A., Brazaitis, G., Campagnaro, T., Ciach, M., Curovic, M., Doerfler, I., Fotakis, D., Govedar, Z., Gregor, K., Gültekin, Y. S., Heilmann-Clausen, J., Hoffmann, J., Hofmeister, J., Jansone, D., Jansons, Ā., Kepfer-Rojas, S., Lachat, T., Lapin, K., Lõhmus, A., Manton, M., Mikac, S., Mikoláš, M., Mohren, F., Nordén, B., Odor, P., Oettel, J., Paillet, Y., Panayotov, M., Roibu, C.-C., Sitzia, T., Svoboda, M., Tanács, E., Trentanovi, G., Vacchiano, G., van der Sluis, T., Zlatanov, T., and Burrascano, S. (2025) Can triad forestry reconcile Europe's biodiversity and forestry strategies? A critical evaluation of forest zoning, Ambio, 54, 632-641, 10.1007/s13280-024-02116-2.
